# Comparison between voltammetric detection methods for abalone-flavoring liquid

**DOI:** 10.1515/biol-2021-0035

**Published:** 2021-04-15

**Authors:** Yan Lv, Xu Zhang, Peng Zhang, Huihui Wang, Qinyi Ma, Xueheng Tao

**Affiliations:** Mechanical Engineering Department of Dalian Polytechnic University, Dalian 116034, China

**Keywords:** abalone-flavoring liquid, voltammetric detection methods, principal component analysis, probabilistic neural network, support vector machine

## Abstract

This article attempts to determine the most accurate classification method for different abalone-flavoring liquids. Three common voltammetric detection methods, namely, linear sweep voltammetry (LSV), cyclic voltammetry (CV), and square-wave voltammetry (SWV), were considered. To compare their classification accuracies of abalone-flavoring liquids, three methods were separately adopted to classify five different abalone-flavoring liquids, using a four-electrode (Au, Pt, Pd, and W) sensor array. Then the data acquired by each method were subject to the principal component analysis (PCA): the first three principal components whose eigenvalues were greater than 1 were extracted from each set of data; the cumulative variance contribution rate and the principal component scores of each method were obtained. The PCA results show that the first three principal components obtained by the CV had the highest cumulative variance contribution rate (91.307%), indicating that the CV can more comprehensively characterize the information of abalone-flavoring liquid samples than the other two methods. According to the principal component scores, compared with those of LSV and SWV, the same kind of samples detected by the CV were highly clustered and the different kinds of samples detected by the CV were greatly dispersed. This indicates that the CV can effectively distinguish between the five abalone-flavoring liquids. Finally, the detection data were further verified through probabilistic neural network and a support vector machine algorithm optimized by genetic algorithm. The results further confirm that the CV is more accurate than the other two methods in the classification of abalone-flavoring liquids. Therefore, the CV was recommended for the classification of abalone-flavoring liquids.

## 1 Introduction

Abalone is a precious and nutritious seafood that is tender and delicious. Instant abalone products are greatly favored by consumers, thanks to their unique flavor, ease of storage, and rich nutrition [1]. The taste of instant abalone products is mainly controlled by precooking in flavoring liquid. Currently, the taste quality of abalone-flavoring liquid is generally subject to sensory evaluation by technicians. The evaluation results are highly subjective, poorly reproducible, and difficult to quantify [2]. In recent years, voltammetric electronic tongue, an electrochemical tool, has widely been used to classify and evaluate the quality of various liquid samples, because of its high sensitivity, repeatability, and reliability.

The voltammetric electronic tongue is often applied with a customary voltammetric detection method. But the detection results may change with specific method. To make a comprehensive and objective evaluation of the object, it is necessary to select the most suitable electrochemical detection method, according to the features of the object [3,4]. Through contrastive experiments, Gholivand et al. [5] compared the effects of cyclic voltammetry (CV), linear sweep voltammetry (LSV), and differential pulse voltammetry (DPV) in detecting bovine serum albumin (BSA). While doing so, they have discovered that the DPV is the most suitable method for BSA detection, and applied the experimental results to clinical treatment. Bikmeev et al. [6] analyzed the difference between LSV, DPV, and square-wave voltammetry (SWV) in the accuracy of classifying the cations in engine oil. Zhang [7] classified honey with DPV, SWV, and CV and proved the superiority of the DPV in honey classification.

It is a difficult task to classify abalone-flavoring liquids, due to the various complex components. This article compares the classification accuracies of LSV, CV, and SWV on five different abalone-flavoring liquids, aiming to select the most suitable detection method for abalone-flavoring liquid.

## 2 Methodology

### 2.1 Materials

To preserve the freshness and aroma of seafood, instant abalone products must be precooked in flavoring liquid, which mainly consists of salt, monosodium glutamate (MSG), and bone broth, plus a few additional ingredients. The mix ratio of these components can be adjusted by the food processor based on consumer needs.

This article compares three voltammetric detection methods on five different abalone-flavoring liquids. The formulas of five flavoring liquids are listed in Table 1. The spices used in our experiments include salt (natural sun-dried salt of China National Salt, GB/T 5461), vinegar (distilled vinegar of Dingfeng, SB/T 10337), MSG (MSG of Meihua, GB/T 8967), and sugar (first-grade white granulated sugar of Hongmian, GB/T 137).

**Table 1:** Formulas of five different abalone-flavoring liquids^a^

Taste of abalone-flavoring liquids	Salt content (g)	MSG content (g)	Vinegar content (mL)	Sugar content (g)
Light	1	0.5	0	0.5
Sweet and fresh	1	2	0	3
Salt and fresh	3	2	0	0
Sour and sweet	1	0.5	2	3
Sour and fresh	1	2	2	0

a Formulas listed in Table 1 refer to the amount of spices in every 100 mL of abalone-flavoring liquids.

The samples to be tested were prepared by weighing the spices according to the formulas in Table 1 and dissolving them in deionized water. Each kind of flavoring liquid was prepared 30 min before the experiment and stored at room temperature (23°C). Right before use, 60 mL of the corresponding flavoring liquid was taken as a sample.

### 2.2 Instruments

The voltammetric electronic tongue detection system used in our experiments consists of a sensor array (Tianjin Aida Hengsheng Technology Development Co. Ltd), a signal excitation and data acquisition device (Shanghai Chenhua Instrument Co., Ltd), and a computer. The sensor array consists of four working electrodes, a reference electrode, and an auxiliary electrode. The working electrodes (diameter: 2 mm) are metal disk electrodes made of gold (Au), platinum (Pt), lead (Pd), and tungsten (W), respectively. The reference and auxiliary electrodes (size: 1 mm × 10 mm) are made of platinum wires. The signal excitation and data acquisition device is a CHI620B electrochemical workstation. The computer is responsible for data processing and analysis. The three parts constitute a complete voltammetric electronic tongue detection system [8]. During operation, the weak signal outputs from the sensor array are collected by the electrochemical workstation and transferred to the computer for processing.

### 2.3 Methods

In the voltammetric electronic tongue detection system, the potential remains constant between the working electrode and the reference electrode, which keeps the voltage stable in the liquid system to excite the current. Then the current on the loop between the working electrode and the auxiliary electrode is taken as the object of detection.

Apart from the conventional pulse voltammetry, multiple ways are involved in generating excitation, namely, DPV, LSV, CV, and SWV [9]. This article attempts to compare the classification effects of LSV, CV, and SWV on abalone-flavoring liquid and select the most suitable method for that liquid.

#### 2.3.1 LSV

**Figure 1:**
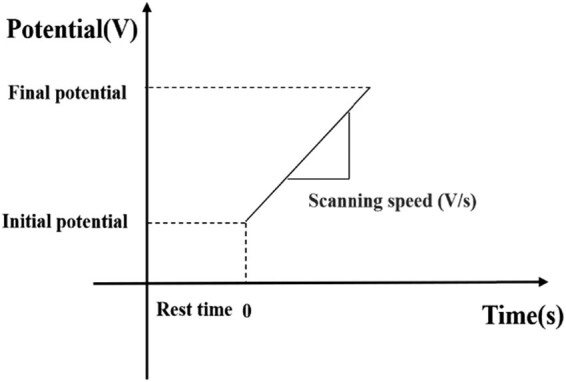
The potential curve applied on the working electrode in the LSV.

In the LSV, a linear voltage is applied between the working electrode and the auxiliary electrode, that is, the electrode potential changes linearly with the applied voltage. Figure 1 illustrates the curve of the potential applied on the working electrode in the LSV. The linear sweep voltammogram can be obtained by recording the variation in the current curve of the working electrode with the potential [10].

Voltammetry is affected by various factors, such as electrolyte, reference electrode, sweep speed, and potential range. The selection of experimental parameters has a great impact on the subsequent operations such as data processing and waveform generation. Through multiple experiments and comparisons, the LSV parameters in our experiments were determined (Table 2).

**Table 2:** Parameters of LSV used in the experiment

Initial potential (V)	Final potential (V)	Sweep rate (V/s)	Sampling interval (V)	Sampling frequency (Hz)	Standing time (s)	Sensitivity range
−0.4	1	0.1	0.001	50	2	e^−5^ to e^−3^

#### 2.3.2 CV

**Figure 2:**
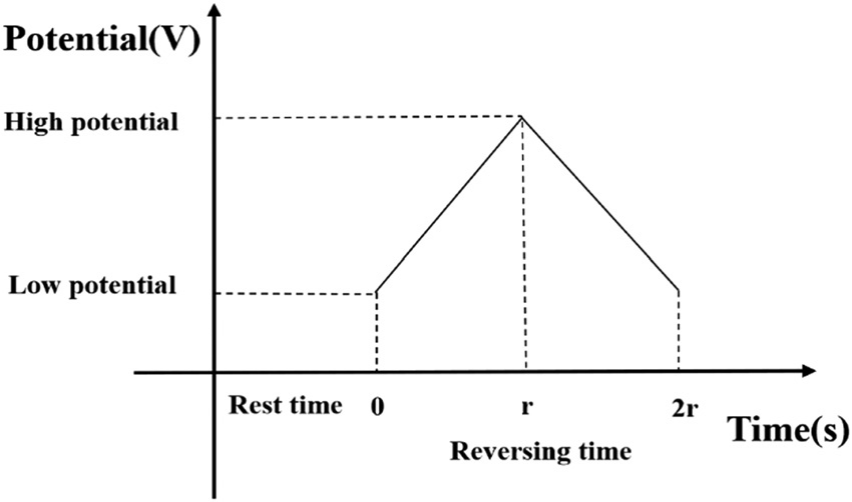
The potential curve applied on the working electrode in the CV.

The CV got its name because it has a reverse sweep operation in addition to the forward sweep of the LSV. Figure 2 shows the change in the potential applied onto the working electrode. It can be seen that the potential curve is in the shape of a triangular wave [10].

Through multiple experiments and comparisons, the CV parameters in our experiments were determined (Table 3).

**Table 3:** Parameters of CV used in the experiment

Initial potential (V)	High potential (V)	Low potential (V)	Sweep rate (V/s)	Sampling interval (V)	Sampling frequency (Hz)	Standing time (s)	Sensitivity range
−0.8	0.6	−0.8	0.1	0.001	50	2	e^−5^ to e^−3^

#### 2.3.3 SWV

**Figure 3:**
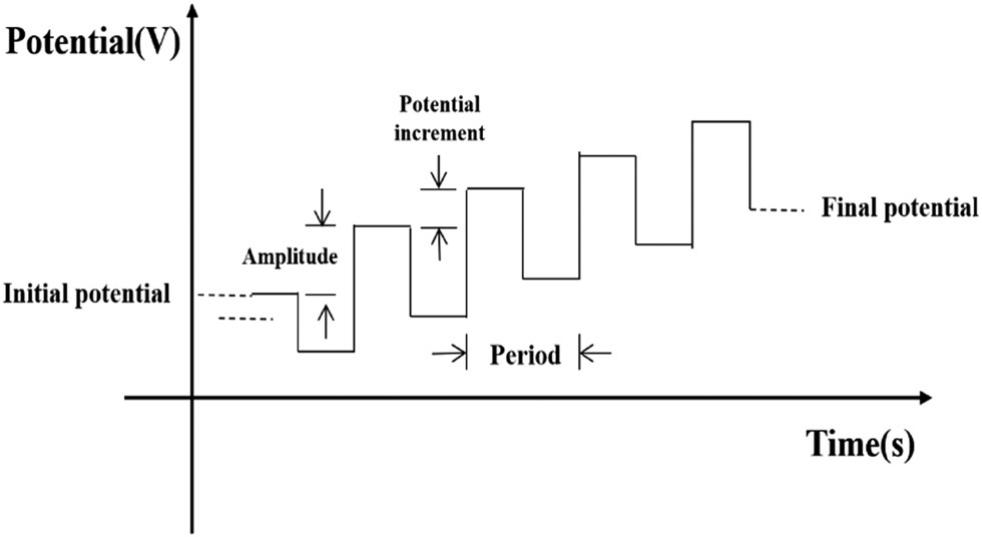
The potential curve applied on the working electrode in the SWV.

Like the CV, every voltammetry method has an excitation signal. As shown in Figure 3, the SWV excitation signal is superimposed from a symmetrical square wave and a stepped voltage. In each cycle, the SWV needs to sample the response current of the working electrode twice, once at the end of the previous pulse and once at the end of the reverse pulse. The net current that equals the difference between the two sampled currents is taken as the input current. Despite being the difference between the two currents, the net current is larger than any of the two sampled currents. As a result, the SWV has a very high sensitivity. The square-wave voltammogram can be obtained from the net current and step voltage [7,10].

The basic parameters of the SWV include the pulse height relative to the step potential and square-wave frequency. Compared with other voltammetric methods, the SWV can complete a voltammogram in a short time. Table 4 presents the setting of SWV parameters in our experiments.

**Table 4:** Parameters of SWV used in the experiment

Initial potential (V)	Final potential (V)	Potential increment (V)	Amplitude (V)	Sampling frequency (Hz)	Standing time (s)	Sensitivity range
−0.8	0.4	0.004	0.025	15	2	e^−5^ to e^−3^

### 2.4 Data collection and preprocessing

During the experiments, three voltammetric methods were separately adopted to test the five different abalone-flavoring liquids in Table 1, using four electrodes (Au, Pt, Pd, and W). Three parallel samples were prepared for each abalone-flavoring liquid. Each sample was repeatedly tested five times.

The peak response current of all sensors under the potential excitation signal was selected as the measured value of one detection. In this way, 75 sets of four-dimensional (4D) data were obtained for each detection method, where 75 refers to five different liquids tested in three parallel samples and repeated five times (75 = 5 × 3 × 5); and 4D refers to the four kinds of signals captured by four working electrodes. From the 75 sets of 4D data for each detection method, the abnormal data were removed by the Dixon method. Then the average of the repeated test values was adopted as the eigenvalue of each sample, producing 15 sets of 4D data. To eliminate the dimensional difference, the data were subject to *Z*-score normalization before statistical analysis [11].

## 3 Results analysis

### 3.1 Principal component analysis (PCA)

PCA is the most popular method for data processing and analysis. The essence of the PCA algorithm is to construct a series of linear combinations of the original variables, aiming to maximize the variance. The linear combinations must be uncorrelated with each other and reflect as much information of the original variables as possible, thereby reducing data redundancy. In the data thus obtained, the first few components contain most of the information in the sample space. Therefore, the entire sample space can be described by the contribution scores of the first few principal components. In addition, the importance of each sample in the principal components can be intuitively illustrated with the score map of these components [12].

**Figure 4:**
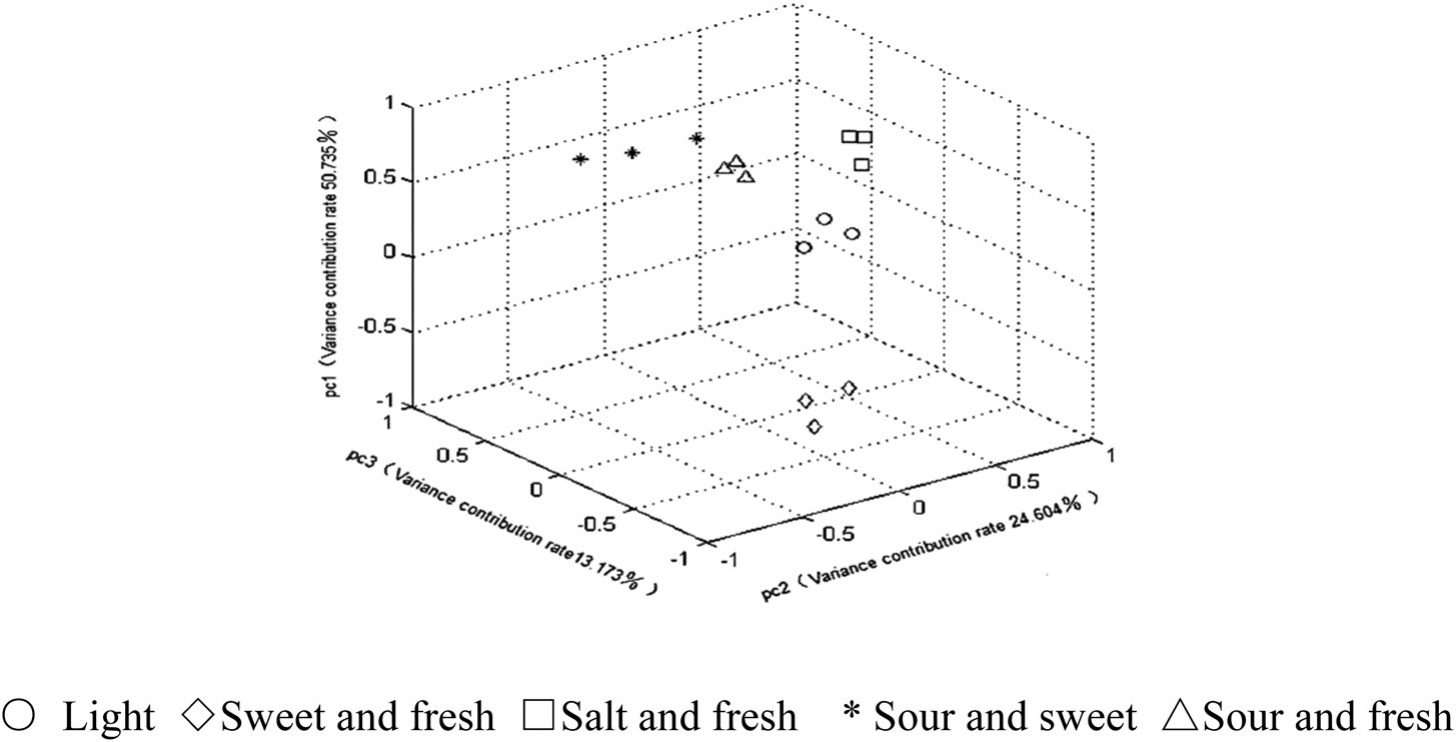
PCA score plots of LSV.

**Figure 5:**
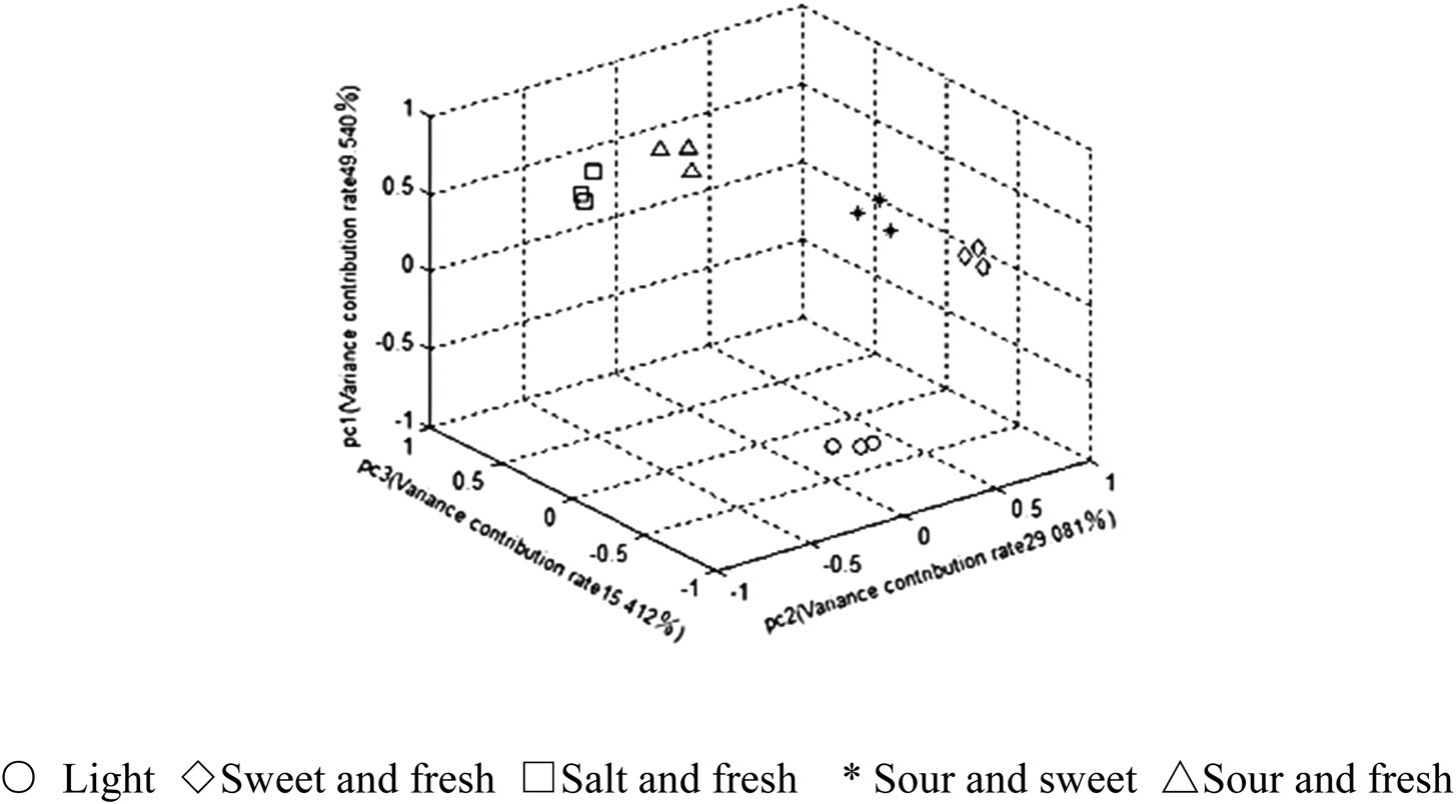
PCA score plots of CV.

**Figure 6:**
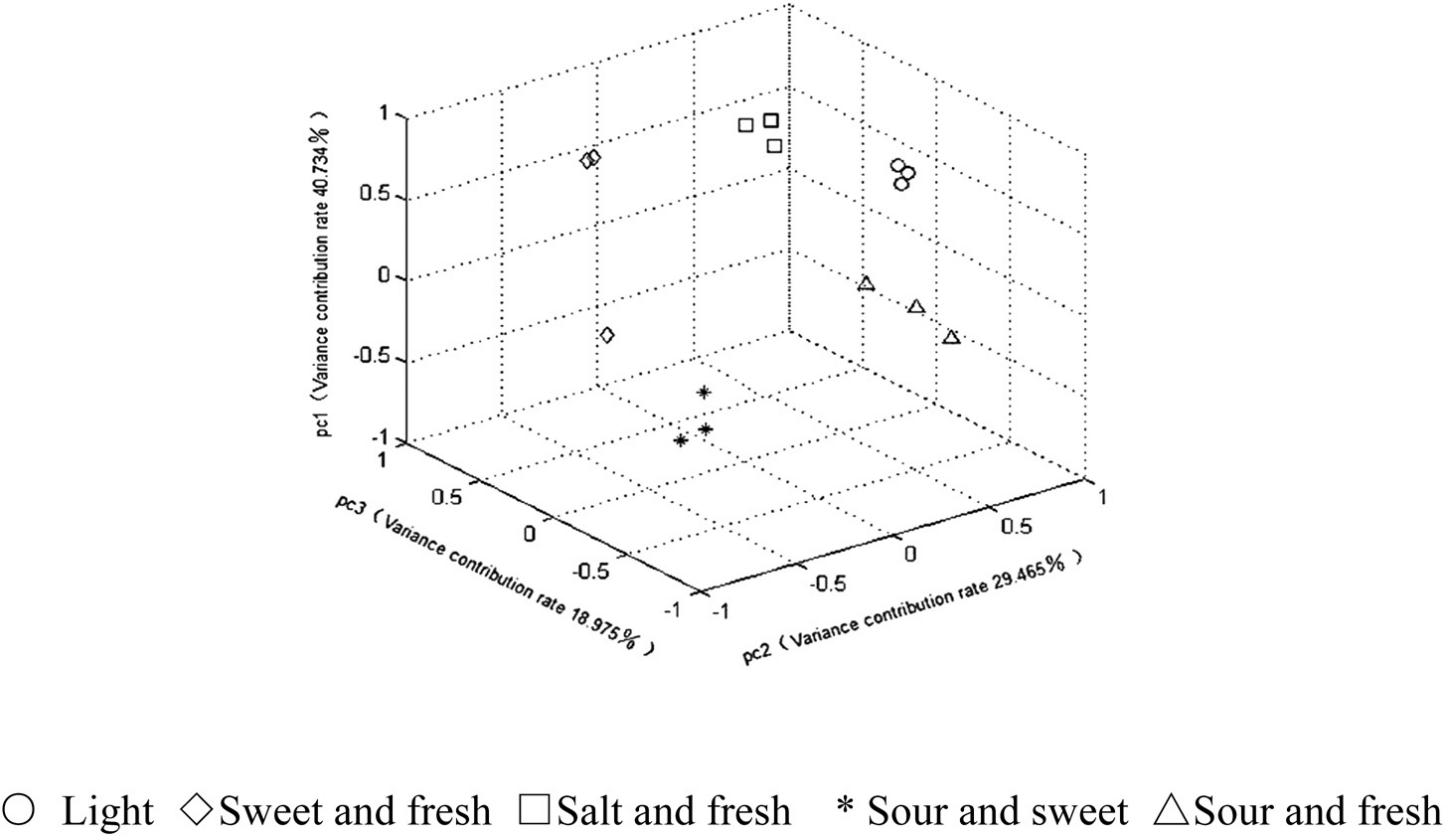
PCA score plots of SWV.

To compare the classification effects of the three detection methods on five different abalone-flavoring liquids, the PCA was performed on the data acquired by each method and the first three principal components whose eigenvalues were greater than 1 were extracted from each set of data. The cumulative variance contribution rate was calculated for the principal components of each method. Then the PCA results are recorded in Table 5, and the principal component scores were obtained as in Figures 4–6.

**Table 5:** PCA results of three detection methods

Detection method	Variance contribution rate of the first principal component (PC1)	Variance contribution rate of the second principal component (PC2)	Variance contribution rate of the third principal component (PC3)	Cumulative variance contribution rate of three principal components (PC1 + PC2 + PC3)
LSV	50.735%	24.604%	13.173%	88.512%
CV	49.540%	29.081%	15.412%	91.307%
SWV	40.734%	29.465%	18.975%	89.174%

As shown in Table 5, the first three principal components obtained by the CV had the highest cumulative variance contribution rate (91.307%). The higher the cumulative variance contribution rate, the stronger the ability of the detection method to retain the information of the original data and classify the samples. Thus, the PCA results indicate that the CV can more comprehensively characterize the information of abalone-flavoring liquid samples than the other two methods.

According to the principal component scores (Figures 4–6), the same kinds of samples detected by the CV were more clustered than those detected by the other two methods, and different kinds of samples detected by the CV were more dispersed than those detected by the other two methods. This further confirms that the CV can effectively distinguish different abalone-flavoring liquids.

### 3.2 Classification results of probabilistic neural network (PNN)

As a popular neural network (NN) for pattern classification, the PNN is a network classifier that separates the decision space within the multidimensional input space by the Bayesian decision rules (minimizing the misclassification risk), based on the statistical principles. The popularity of the PNN as a pattern classifier is attributable to its simple learning process, fast training speed, accurate classification, and good fault tolerance [13].

In this research we have employed PNN to verify the classification accuracy of the three detection methods on different abalone-flavoring liquids. From the 75 sets of 4D data for each detection method, 50 sets were randomly selected and organized into a training set to build the PNN model and the remaining 25 sets were organized into a test set to check the classification accuracy of the model.

The PNN model contains four input layer nodes and five output layer nodes. The smoothing parameter was set to 1.5. After the network training on the training set, the data in the test set were distributed to different classes.

Table 6 compares the PNN classification results of three detection methods. In the CV, LSV, and SWV test sets, two sets, three sets, and two sets of data were incorrectly allocated to other classes. Therefore, the classification accuracies of the CV, LSV, and SWV were 92, 88, and 92%, respectively. Similar to the PCA results, the PNN-based verification proves that the CV and SWV have better detection effect than the LSV.

**Table 6:** PNN results of three detection methods

Detection method	Number of training samples	Number of test samples	Number of correctly identify samples	Recognition accuracy (%)
LSV	50	25	22	88
CV	50	25	23	92
SWV	50	25	23	92

### 3.3 Classification results of support vector machine (SVM) optimized by genetic algorithm (GA)

The GA, a search algorithm for optimal solution, is inspired by the theory of evolution and genetics in computational mathematics. After initializing the population by genetic coding, the GA performs genetic operations on the individuals in the population based on their environmental fitness, such that only the fittest can survive. From the perspective of optimal search, genetic operations make the solutions of the problem evolve from generation to generation and gradually approach the optimal solution [14]. SVM is a pattern recognition technique based on statistical learning. The technique can be applied to data analysis, pattern recognition, classification, and regression analysis.

From the 75 sets of 4D data for each detection method, 50 sets were randomly selected and organized into a training set to build the SVM model, and the remaining 25 sets were organized into a test set to check the classification accuracy of the model. The key to SVM lies in the selection and construction of the kernel function. The most commonly used kernel function is the radial basis function (RBF), which can effectively process samples of any size. This article selects the SVM algorithm with RBF. The penalty factor *c* and kernel function parameter *g* of the algorithm were optimized by GA [15]. The optimal results are shown in Table 7.

**Table 7:** Parameter optimization results of SVM by GA

Detection method	Penalty factor *c*	Kernel function parameter *g*
LSV	12.34	1.2
CV	15.76	1.2
SWV	16.37	1.4

The GA-optimized SVM algorithm was adopted to verify the classification accuracies of the data obtained by three detection methods. The verification results (Table 8) show that the GA-optimized SVM algorithm achieved the best accuracy on the data obtained by the CV, which agrees with the PCA results.

**Table 8:** GA-optimized SVM algorithm recognition results of three detection methods

Detection method	Number of training samples	Number of test samples	Number of correctly identify samples	Recognition accuracy (%)
LSV	50	25	23	92
CV	50	25	24	96
SWV	50	25	23	92

The above analysis shows that CV outperforms LSV and SWV in the coverage of sample information and classification accuracy. Therefore, CV was recommended for the classification of abalone-flavoring liquids.

## 4 Conclusions

Considering the complex compositions of abalone-flavoring liquid, three common voltammetric detection methods were selected to test five different abalone-flavoring liquids, using a sensor array of four electrodes (Au, Pt, Pd, and W). The classification accuracies of three methods on the five liquids were compared to select the most suitable method for the detection of abalone-flavoring liquid. The detection data obtained by each method were preprocessed in two steps: the abnormal data were removed by the Dixon method and the remaining data were subject to *Z*-score normalization. After that the normalized data were statistically analyzed by the PCA, PNN, and GA-optimized SVM.

(1) The PCA results show that the first three principal components obtained by the CV had the highest cumulative variance contribution rate (91.307%), indicating that the CV can characterize the information of abalone-flavoring liquid samples more comprehensively than the other two methods. According to the principal component scores, compared with those of LSV and SWV, the same kinds of samples detected by the CV were highly clustered and the different kinds of samples detected by the CV were greatly dispersed. This further confirms that the CV can effectively distinguish between different abalone-flavoring liquids.
(2) The verification results of the PNN and GA-optimized SVM also prove that the CV is more accurate than the other two methods in the classification of abalone-flavoring liquids. Therefore, the CV was recommended for the classification of abalone-flavoring liquids.
